# Prioritizing Community in Research Decision-Making Through Partnership

**DOI:** 10.1093/geroni/igab046.1824

**Published:** 2021-12-17

**Authors:** Nicole Marrone, Maia Ingram, Aileen Wong, Rosie Piper, Sonia Colina, Scott Carvajal, Laura Coco

**Affiliations:** 1 University of Arizona, Tucson, Arizona, United States; 2 Mariposa Community Health Center, Nogales, Arizona, United States

## Abstract

In behavioral intervention research, taking a community-based participatory research approach enhances recruitment and retention while facilitating the transfer of research findings into social change. Successes with recruitment and retention are secondary to enacting fundamental principles of trust, reciprocity, cultural humility, empowerment, and respect. This presentation will describe a longitudinal clinical trial in a Southwest borderlands community, Oyendo Bien. The study was co-developed and implemented with community partnership throughout the research process. Dyads were recruited to participate in a community-delivered group education and support program addressing hearing loss for Spanish-speakers age 50+ years (n=132 participants randomized). We highlight the critical role that community health workers (promotoras) held as members of the research team. Furthermore, we describe an innovative approach for language mediation that integrates and empowers community participation. This presentation will include examples of lessons learned from the community in collaborating to conduct research in a way that truly serves.

